# Novel mutations of the X-linked genes associated with early-onset high myopia in five Chinese families

**DOI:** 10.1186/s12920-023-01665-x

**Published:** 2023-09-25

**Authors:** Feiyin Zi, Zhen Li, Wanyu Cheng, Xiaoyu Huang, Xunlun Sheng, Weining Rong

**Affiliations:** 1 Clinical Medical College, Ningxia Medical University, Yinchuan, 750001 China; 2 Department of Ophthalmology, Ningxia Eye Hospital, People’s Hospital of Ningxia Hui Autonomous Region, Third Clinical Medical College of Ningxia Medical University, 936 Huanghe East Road, Jinfeng District, Yinchuan, 750001 China; 3 Gansu Aier Ophthalmiology and Optometry Hospital, 1228 Guazhou Road, Qilihe District, Lanzhou, 730050 China

**Keywords:** Early-onset high myopia, X-linked recessive inheritance, Genetic variant, Clinical phenotype

## Abstract

**Purpose:**

To report novel pathogenic variants of X-linked genes in five Chinese families with early-onset high myopia (eoHM) by using whole-exome sequencing and analyzing the phenotypic features.

**Methods:**

5 probands with X-linked recessive related eoHM were collected in Ningxia Eye Hospital from January 2021 to June 2022. The probands and their family members received comprehensive ophthalmic examinations,and DNA was abstracted from patients and family members. Whole-exome sequencing was performed on probands to screen the causative variants, and all suspected pathogenic variants were determined by Sanger sequencing and co-segregation analysis was performed on available family members. The pathogenicity of novel variants was predicted using silico analysis and evaluated according to ACMG guidelines. RT-qPCR was used to detect differences in the relative mRNAs expression of candidate gene in mRNAs available with the proband and family members in the pedigree 2. The relationship between genetic variants and clinical features was analyzed.

**Results:**

All probands were male, and all pedigrees conformed to an X-linked recessive inheritance pattern. They were diagnosed with high myopia at their first visits between 4 and 7 years old. Spherical equivalent ranged between − 6.00D and − 11.00D.The five novel hemizygous variants were found in the probands, containing frameshift deletion variant c.797_801del (p.Val266Alafs*75) of *OPN1LW* gene in the pedigree 1, nonsense variant c.513G > A (p.Trp171Ter)of *RP2* gene in the pedigree 2, missense variant c.98G > T (p.Cys33Phe) of *GPR143* gene in the pedigree 3, frameshift deletion variant c.1876_1877del (p.Met626Valfs*22) of *FRMD7* gene in the pedigree 4 and inframe deletion variant c.670_ 675del (p.Glu192_ Glu193del) of *HMGB3* gene in the pedigree 5. All variants were classified as pathogenic or likely pathogenic by the interpretation principles of HGMD sequence variants and ACMG guidelines. In family 2, RT-qPCR showed that the mRNA expression of *RP2* gene was lower in the proband than in other normal family members, indicating that such variant caused an effect on gene function at the mRNA expression level. Further clinical examination showed that pedigrees 1, 2, 3, and 4 were diagnosed as X-linked recessive hereditary eye disease with early-onset high myopia, including quiescent cone dysfunction, retinitis pigmentosa, ocular albinism, and idiopathic congenital nystagmus respectively. The pedigree 5 had eoHM in the right eye and ptosis in both eyes.

**Conclusion:**

In this paper,we are the first to report five novel hemizygous variants in *OPN1LW, RP2, GPR143, FRMD7, HMGB3* genes are associated with eoHM. Our study extends the genotypic spectrums for eoHM and better assists ophthalmologists in assessing, diagnosing, and conducting genetic screening for eoHM.

## Introduction

High myopia(HM) is defined as less than or equal to -6.00 diopters or an ocular axis length of at least 26 mm [1]. According to the age of onset, high myopia is classified into late-onset high myopia (loHM), which occurs at post-school age, and early-onset high myopia (eoHM), which occurs at preschool age. Available evidences suggest that both environmental and genetic factors are involved in high myopia development and progression, of which the environmental factors include proximity homework, reading habits, heavier school burden, and less outdoor activities [2]. Numerous genetic studies have shown that eoHM differs from loHM. The onset of eoHM occurs at preschool age (< 7 years), which is minimally influenced by environmental factors, and is primarily determined by genetic factors. Therefore, eoHM is an ideal model for studying the pathogenesis of high myopia and a unique resource for finding genes associated with high myopia. eoHM can be classified as a simple type (non-syndromic) manifestation with high myopia alone and as a syndromic manifestation complicated with other eye diseases or systemic abnormalities [3]. As reported published by Holden [4] et al. in the World Health Organization (WHO) in 2015, the prevalence of myopia in China is close to 50%, with 15.73% of school-age children suffering from high myopia, much higher than in Europe, North and South America. Complications such as choroidal neovascularization (CNV) and macular degeneration associated with high myopia, which can lead to Permanent visual impairment and even blindness, have become one of the major leading causes of blindness in young and middle-aged people over 30 years of age [5] and can cause severe quality of life (functional, psychological and economic) reduction.

To date, 17 pathogenic genes causing non-syndromic high myopia have been identified, including *ZNF644* [6], *SCO2* [7], *SLC39A5* [8], *CCDC111* [9], *P4HA2* [10], *BSG* [11], *CPSF1* [12], *NDUFAF7* [13], *TNFRSF21* [14], *XYLT1*^[ 15]^, *DZIP1* [15], *LRPAP1* [16], *CTSH* [17], *LEPREL1* [17], *LOXL3* [18], *ARR3* [19] and *OPN1LW* [20], but these gene variations explain only a small part of the pathogenesis of eoHM. Recent studies have shown that approximately 39% of early-onset high myopia may be caused by mutations in genes associated with a number of other inherited eye diseases [21]. For example, mutations in *COL2A1* and *COL11A1* genes can cause Stickler syndrome [22], mutations in *EYS* gene cause RP [23], *TSPAN12* and *FZD4* are common causative genes for FEVR [24], and high myopia is also an early clinical phenotype of inherited retinal diseases such as cone rod dystrophy(CORD) and gyrate atrophy of the choroid and retina(GA).

High myopia, to different degrees, is a prominent feature of several X-linked disorders, to the extent that its presence often suggests the possibility of some X-linked inherited retinal diseases [25]. However, fewer studies have analyzed the phenotype of eoHM patients with variations in X-linked genes. In this paper, we describe five patients with eoHM carrying novel variations in X-linked genes from five Chinese families and discuss the phenotypic characteristics of individuals with different genotypes, providing reliable molecular diagnosis for the X-linked recessive eoHM and offering options for eugenics for these kinds of family members.

## Materials and methods

### Ethical approval

This study was approved by the Ethics Committee of People’s Hospital of Ningxia Hui Autonomous Region (Approval No. 2022-LL-022), and it was conducted in accordance with the 1975 Declaration of Helsinki guidelines.Written informed consent was obtained from all included subjects or their legal guardians before participation.

### Subjects and clinical evaluations

The probands who received the initial diagnosis as eoHM and their family members were collected in Ningxia Eye Hospital, People’s Hospital of Ningxia Hui Autonomous Region from January 2021 to June 2022 for both genetic and clinical tests. Patients were included if they had high myopia (spherical equivalent≤-6.00D or axial length ≥ 26 mm)at preschool age (< 7 years). In addition to high myopia, the probands also might have ,or not, other ocular or systemic abnormalities. The relevant ophthalmologic examinations were completed for all probands and their family members, including best corrected visual acuity (BCVA), chromoptometry(5th edition color blindness examination chart, Ziping YU), slit lamp microscopy, dilated fundus examination with photography(Optos DaytonaP200T), optical coherence tomography (OCT) (HD-OCT4000, Carl Zeiss Meditec, USA) and fundus autofluorescence(FAF). The present medical history, previous medical history, personal history, family history and marital history of the pedigrees were inquired and recorded in detail, and the pedigree charts were drawn.

### Whole-exome sequencing

5 ml of peripheral venous blood was collected from each participant,and genomic DNA was extracted using Qiamp Blood Mini Kit DNA extraction kit (Qiagen, Germany). Whole-exome sequencing was selectively performed on probands. Exome was captured by Agilent SureSelect exon capture kit and sequencing was served with a highthroughput sequencer (Illumina, HiSeq Xten) with a depth of 100 ×.The raw sequencing data were processed by Illumina basecalling Software 1.7 and subsequently compared with the NCBI human genomic DNA reference sequence (NCBI build 37.1). Single nucleotide variation (SNV) was analyzed by SOAP software (http://soap.genomics.org.cn) while insertion and deletion variants (Indel) were analyzed by BWA software (bio-bwa.sourceforge.net/) to obtain all the variants occurring in the DNA sequences in the samples. High-frequency variations with minimum allele frequency (MAF) > 1% were filtered from the database (db135). The variants have no effect on the structure and function of the protein were filtered out. Sanger validation was used to exclude false positives for candidate pathogenic variants, and co-segregation of genotypes and phenotypes was further verified in the normal pedigree members.

### In silico analysis

The pathogenicity of novel variants was assessed according to the *Standards and Guidelines for Interpretation of Sequence Variants* issued by the American College of Medical Genetics and Genomics (ACMG) in 2015. MAF less than 0.005 was used as the criteria to exclude benign variants by reference to the databases, including the normal population gene frequency 1,000 genomes (1,000 genomes), EXAC (The Exome Aggregation Consortium), and EXAC-EAS (about 4,000 East Asians data under EXAC). The effects of mutation sites on protein function were predicted by publicly available servers such as SIFT (https://grch37.ensembl.org/Tools/VEP), Polyphen-2 (http://genetics.bwh.harvard.edu/pph2/) and Mutation Taster (http://mutationtaster.org/). When all predictions were pathogenic, variants were classified as possibly pathogenic in combination with other evidences. Frameshift variants, nonsense variants, and variants with experimental evidences of causing loss of protein function were classified as pathogenic variants. The online analysis tool Multalin (http://multalin.toulouse.inra.fr/multalin/multalin.html) was used for conservativeness analysis of variant loci. The spatial structure of these wild type and mutated proteins were modeled by Alpha Fold 2 and Misssense 3D, then were aligned with PyMOL 2.3 software.

### Analysis of relative mRNA expression in candidate gene

Real-time quantitative PCR (RT-qPCR) was used to measure the relative expression of *RP2* gene mRNA in pedigree 2. Total RNA was isolated from blood samples using the PAXgene Blood RNA Kit (Qiagen #762,174), followed by determination of RNA concentration and quality by Qubit® 3.0 Fluorometer (Life Technologies, USA) and Nanodrop One Spectrophotometer (Thermo Fisher Scientific Inc., USA). The integrity of total RNA was assessed using an Agilent 2100 Bioanalyzer (Agilent Technologies Inc., USA). cDNA synthesis was performed on 1 μg of RNA using the Hifair® II 1st Strand cDNA Synthesis Kit (gDNA digester plus) (Yeason Biotech, 11121ES50). Intron-crossing primers for the *RP2* gene were designed. Forward primer-AGAGACGGAAGGCTGACAAG and reverse primer-CAGGTAAGCGACC TACTGTTT. The RT-qPCR reaction system was prepared according to the NovoStart® SYBR qPCR SuperMix Plus kit. The 2-ΔΔCt method was used to calculate the relative mRNA expression of *RP2* gene in the patient and family members, which was normalized by the Ct value of the internal reference GAPDH gene.The proband and family members were tested twice, and the data quality was qualified.

## Results

**Table 1:** Clinical manifestations of 5 probands with early onset high myopia (F)

		age(y)	SE(D)		BCVA		AL (mm)	
			R	L	R	L	R	L
F1	male	6	−6.00	−6.00	0.6	0.6	24.72	24.57
F2	male	5	−8.00	−9.50	0.8	0.6+	25.26	25.38
F3	male	5	−9.00	−11.00	0.15	0.15	26.32	26.69
F4	male	8	−5.00	−6.25	0.6+	0.6+	25.37	25.96
F5	male	12	−10.25	−3.50	0.5+	1.0-	27.58	24.71

SE: spherical equivalent;R:Right eye;L:Left eye;AL:axial lengths

**Table 2:** Genetic test results of 5 probands with early onset high myopia (F)

	F1	F2	F3	F4	F5
Gene	*OPN1LW*	*RP2*	*GPR143*	*FRMD7*	*HMGB3*
Nucleotide	c.797_801del	c.513G > A	c.98G > T	c.1876_1877del	c.670_675del
Amino acid	p.Val266Alafs*75	p.Trp171Ter	p.Cys33Phe	p.Met626Valfs* 22	p.Glu192_Glu193del
Chromosome	chrX: 153,421,821- 153,421,825	chrX: 46,713,321	chrX: 9,733,760	chrX: 131,212,168- 131,212,169	chrX: 150,156,358- 150,156,363
Exon	exon5	exon2	exon1	exon12	exon5
SIFT			D		
Polyphen2_HDIV			D		
Polyphen2_HVAR			D		
LRT		D	D		
Mutation Assessor			M		
REVEL			0.869		
phyloP100way		0.829	5.298		
phastCons100way		0.866	1		
FATHMM			D		
CADD		5.116	3.806		
PROVEAN			D		
Mutation Taster		A	D		
GERP++		5.62	4.51		

### Pedigree 1

**Fig. 1:**
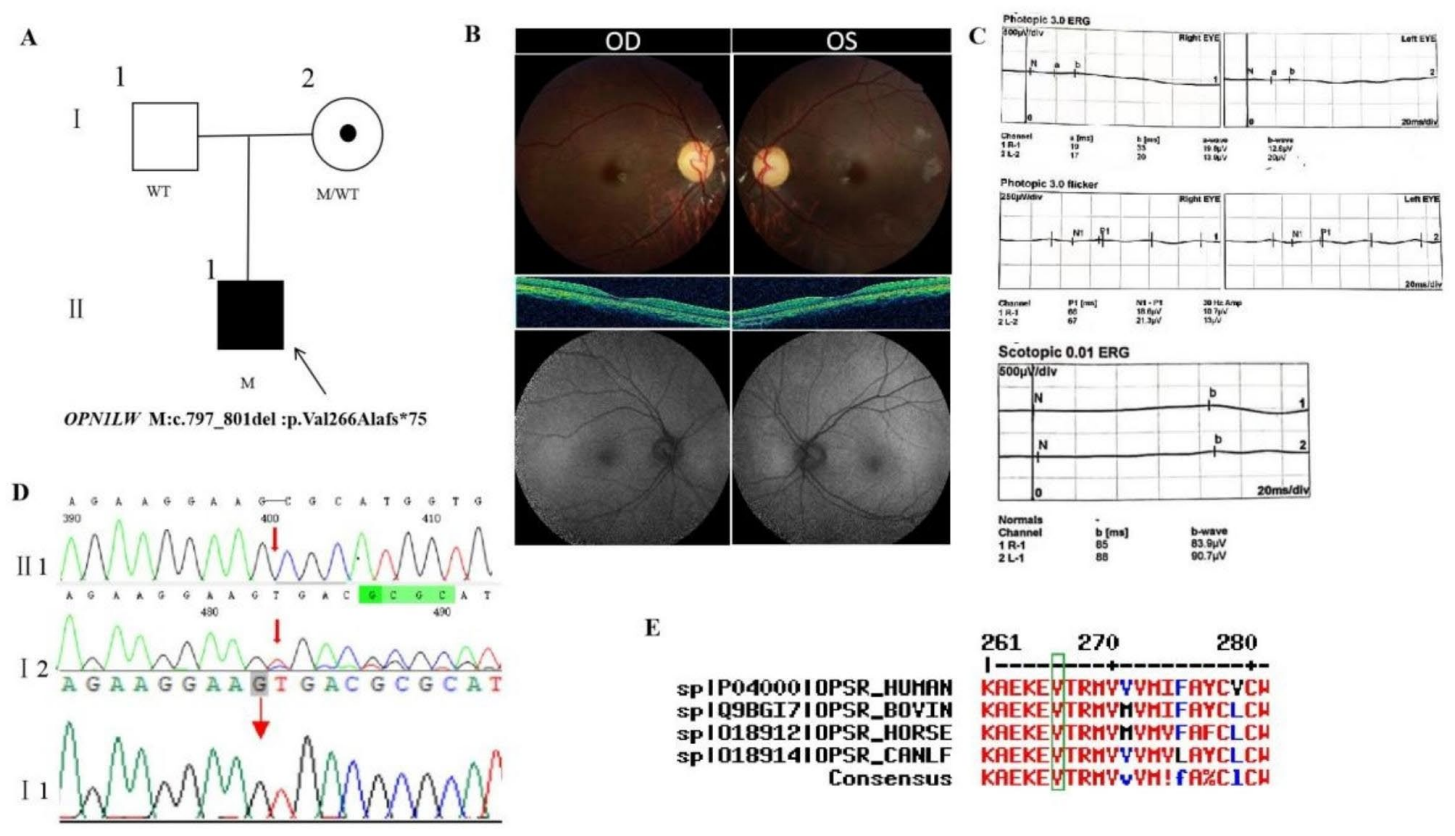
Sequence analysis and clinical examination of the family 1: (**A**) Pedigree of the family 1:The filled black symbol represents the affected member, a central dot denotes carriers, and the arrow denotes the proband. (**B**) The fundus of both eyes: macular OCT of both eyes suggested thinning of the macular fovea and autofluorescence in both eyes suggested a slightly wider range of hypofluorescence in the macula. (**C**) ERG showed significantly decline in cone function and normal rod function in both eyes. (**D**) Sequence chromatograms of identified mutations. (**E**) The homology of amino acid sequences between human *OPN1LW* and other species. The amino acid at position 266 is highly conserved among species, and the mutated residue 266 is boxed and indicated

The proband, male, was 6 years old. His parents noticed the child had poor vision in both eyes. His parents denied a family history of hereditary disease and consanguineous marriage history (Fig. 1A). He had dyserythrochloropsia and BCVA of 0.6 in both eyes (Table 1). The anterior segment was normal,and fundus with no reflection in the macular fovea. OCT showed thinning of the macular fovea and autofluorescence showed a slightly wider range of hypofluorescence in the macula area (Fig. 1B). ERG recording revealed significantly decline in cone function (3.0 ERG b-wave was severely decreased in photopic adaptation and 3.0 flash ERG was severely decreased in photopic adaptation) and normal rod function in both eyes (Fig. 1C). The proband carried a hemizygous frameshift variant c.797_801del (p.Val266Alafs*75) in *OPN1LW* gene on chromosome X. His mother was normal and carried the same heterozygous variant of the *OPN1LW* gene by Sanger sequencing (Fig. 1D) (Table 2), suggesting co-segregation of genotype and clinical phenotype (PP1_Supporting). The frameshift variant has not been previously reported and was also not detected in the East Asian Population Database (ExAC_ EAS) (PM2_Supporting). The truncate mutations would result in the premature termination of polypeptide chain synthesis, and most of the proteins produced were inactive or lost their normal function (PVS1_Very Strong). The c.797_801del variant caused a frameshift starting with coding Valine266, changed this amino acid to an Alanine residue, and created a premature stop codon at amino acid position 75 in the new open reading frame downstream from the deletion, denoted p.Val266Alafs*75. What is more, the amino acid at position 266 was highly conserved among different species by proteomic conservation analysis (Fig. 1E), indicating that the variant of this site is more likely to affect the structure and function of OPN1LW protein (PP3_Supporting). The proband was finally diagnosed as quiescent cone dysfunction with early-onset high myopia in both eyes (PP4). Therefore, the hemizygous frameshift variant c.797_801del (p.Val266Alafs*75) of the *OPN1LW* gene was the pathogenic variant of pedigree 1 according to ACMG guideline (PVS1 + PM2 + PP3 + PP4 + PP1).

### Pedigree 2

**Fig. 2:**
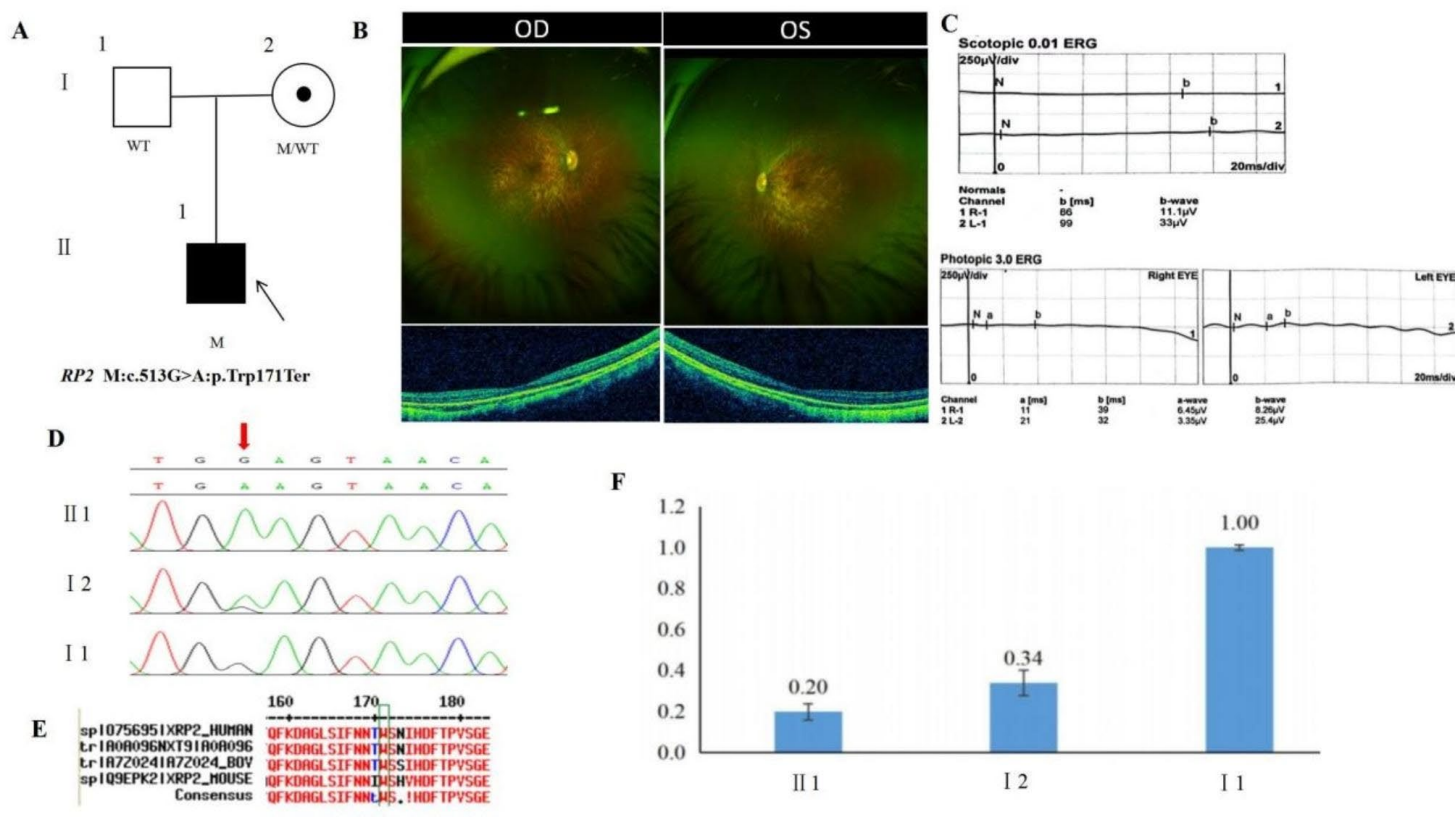
Sequence analysis and clinical examination of the family 2: (**A**) Pedigree of the family 2: The filled black symbol represents the affected member, a central dot denotes carriers, and the arrow denotes the proband. (**B**) The fundus of both eyes: fundus tessellation with macular atrophy, the peripheral retina did not show obvious bone-spicule pigmentation; OCT of both eyes showed thinning of the outer layer of the macula and the disappearance of light reflection signal in the ellipsoid zone and chimeric zone of fovea centralis. (**C**) Scotopic and photopic ERG responses were severe declined in both eyes (**D**) Sequence chromatograms of identified mutations. (**E**) The homology of amino acid sequences between human *RP2* and other species. The amino acid at position 171 is highly conserved among species, the mutated residue 171 is boxed and indicated. (**F**) The mRNA expression of *RP2* gene available with the samples was 0.20 in proband (II:1), 0.34 in mother(I:2), and 1.00 in father (I:1)

The proband is male, 5 years old. His parents found that the child had poor night vision in both eyes for 1 year. His parents denied consanguineous marriage history (Fig. 2A). BCVA was 0.8 on the right eye and 0.6 + on the left eye (Table 1). He had exotropia and the normal anterior segment. Fundus tessellation with macular atrophy, the peripheral retina did not show obvious bone-spicule pigmentation (Fig. 2B). OCT showed thinning of the outer layer of the macula and the disappearance of light reflection signal in the ellipsoid zone and chimeric zone of fovea centralis (Fig. 2B). Scotopic and photopic ERG responses were severe declined in both eyes(Fig. 2C). The proband carried a hemizygous nonsense variant c.513G > A(p.Trp171Ter) in *RP2* gene on chromosome X. His mother was normal and carried the same heterozygous variant of the *RP2* gene by Sanger sequencing (Fig. 2D) (Table 2), suggesting co-segregation of genotype and clinical phenotype (PP1_Supporting). The frameshift variant has not been previously reported and was also not detected in the East Asian Population Database (ExAC_ EAS) (PM2_Supporting). The nonsense variant, c.513G > A, was predicted to generate a premature termination codon at residue 171, and the variant would result in NMD(nonsense mediated decay), which allows most of the mutated transcripts to be eliminated before translation (PVS1_Very Strong). The amino acid at position 171 was highly conserved among different species by proteomic conservation analysis (Fig. 2E), indicating that the variant of this site is more likely to affect the structure and function of RP2 protein (PP1_Supporting). The variant was predicted to be deleterious and pathogenic by several software such as LRT, phyloP100way, phastCons100way, CADD and Mutation Taster (PP3_Supporting). The proband was finally diagnosed as retinitis pigmentosa with early-onset high myopia in both eyes. Therefore, the hemizygous frameshift variant c.513G > A(p.Trp171Ter) of the *RP2* gene was more likely the pathogenic variant of pedigree 2 according to ACMG guideline (PVS1 + PM2 + PP3 + PP1). Nonsense-mediated mRNA decay (NMD) is expected to eliminate RP2 protein expression (including truncated polypeptides) from mutant alleles.Therefore, we used RT-qPCR to detect the expression of *RP2* gene transcript levels in the proband and normal family members. The results showed that the mRNA expression of *RP2* gene available with the samples was 0.20 in the proband (II:1), 0.34 in the mother(I:2), and 1.00 in the father (I:1) (Fig. 2F), indicating that most of the mutated transcripts were eliminated before translation due to nonsense-mediated mRNA decay.

### Pedigree 3

**Fig. 3:**
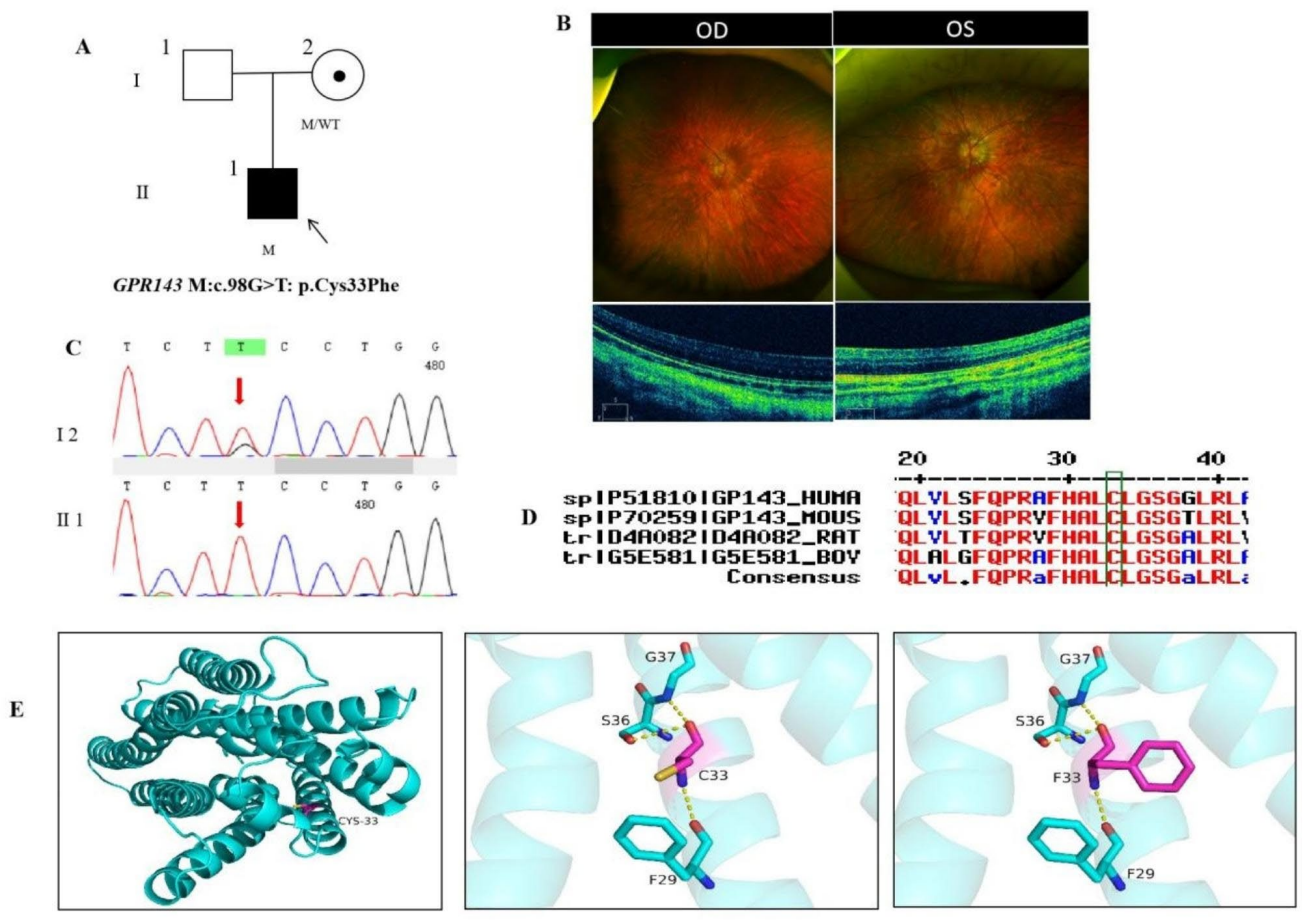
Sequence analysis and clinical examination of the family 3: (**A**) Pedigree of the family 3:The filled black symbol represents the affected member, a central dot denotes carriers, and the arrow denotes the proband. (**B**) The fundus of both eyes: the fundus is orange with pigment loss, parapapillary atrophy around the disc and macular phyoplasia with no reflection; Fovea centralis structure was not observed on OCT. (**C**) Sequence chromatograms of identified mutations. (**D**) The homology of amino acid sequences between human GPR143 and other species. The amino acid at position 33 is highly conserved among species,and the mutated residue 33 is boxed and indicated. (**E**) The protein structure suggested that p. Cys33Phe mutation resulted in the replacement of the cysteine at site 33 with a large, nonpolar uncharged phenylalanine

The proband, male, was 5 years old. His parents complained that the child blinked frequently,and denied family history and consanguineous marriage history (Fig. 3A). He had photophobia, dyserythrochloropsia, horizontal nystagmus, ptosis, esotropia, and inferior eyelids trichiasis with normal skin and hair color. The anterior segment was normal and BCVA of 0.15 in both eyes (Table 1).The fundus is orange with pigment loss, parapapillary atrophy around the disc and macular phyoplasia with no reflection (Fig. 3B). Fovea centralis structure was not observed on OCT. The proband carried a hemizygous missense variant c.98G > T(p.Cys33Phe) in *GPR143* gene on chromosome X and his mother was normal with the same heterozygous variant of the *GPR143* gene by Sanger sequencing (Fig. 3C) (Table 2), suggesting co-segregation of genotype and clinical phenotype (PP1_Supporting). The missense variant has not been previously reported and was also not detected in the East Asian Population Database (ExAC_ EAS) (PM2_Moderate), the amino acid at position 33 was highly conserved among different species (Fig. 3D). The substitution, c.98G > T, which resulted in the amino acid change from Cysteine to Phenylalanine at residue 33(p.Cys33Phe) and four software predictions of SIFT, PolyPhen-2, PROVEAN and Mutation Taster indicated the deleterious impact of this variant (PP3_Supporting) (Table 2). According to the grading criteria for pathogenic variants as described in the ACMG guidelines, c.98G > T (p.Cys33Phe) as a novel missense variant causing amino acid changes was classified as pathogenic moderate (PM5).The proband was finally diagnosed as X-linked recessive ocular albinism (OA1) with early-onset high myopia in both eyes. The hemizygous missense variant c.98G > T(p.Cys33Phe) of the *GPR143* gene was more likely the pathogenic variant of pedigree 3 according to ACMG guideline (PM5 + PM2 + PP3 + PP1). The protein structure suggested that the N atom of the wild-type protein, which is a polar uncharged Cysteine at residue 33, formed a hydrogen bond with the uncharged nonpolar Phenylalanine O atom at residue 29, and the Cysteine O atom formed hydrogen bonds with SER36 and G37, the variant c.98G > T(p.Cys33Phe) resulted in the replacement of the Cysteine at site 33 with a large, nonpolar uncharged Phenylalanine, and the variant didn’t alter amino acid interactions, but the substitution of the Cysteine sulfhydryl functional group by the phenyl ring might result in altered protein structure and function after mutation (Fig. 3E).

### Pedigree 4

**Fig. 4:**
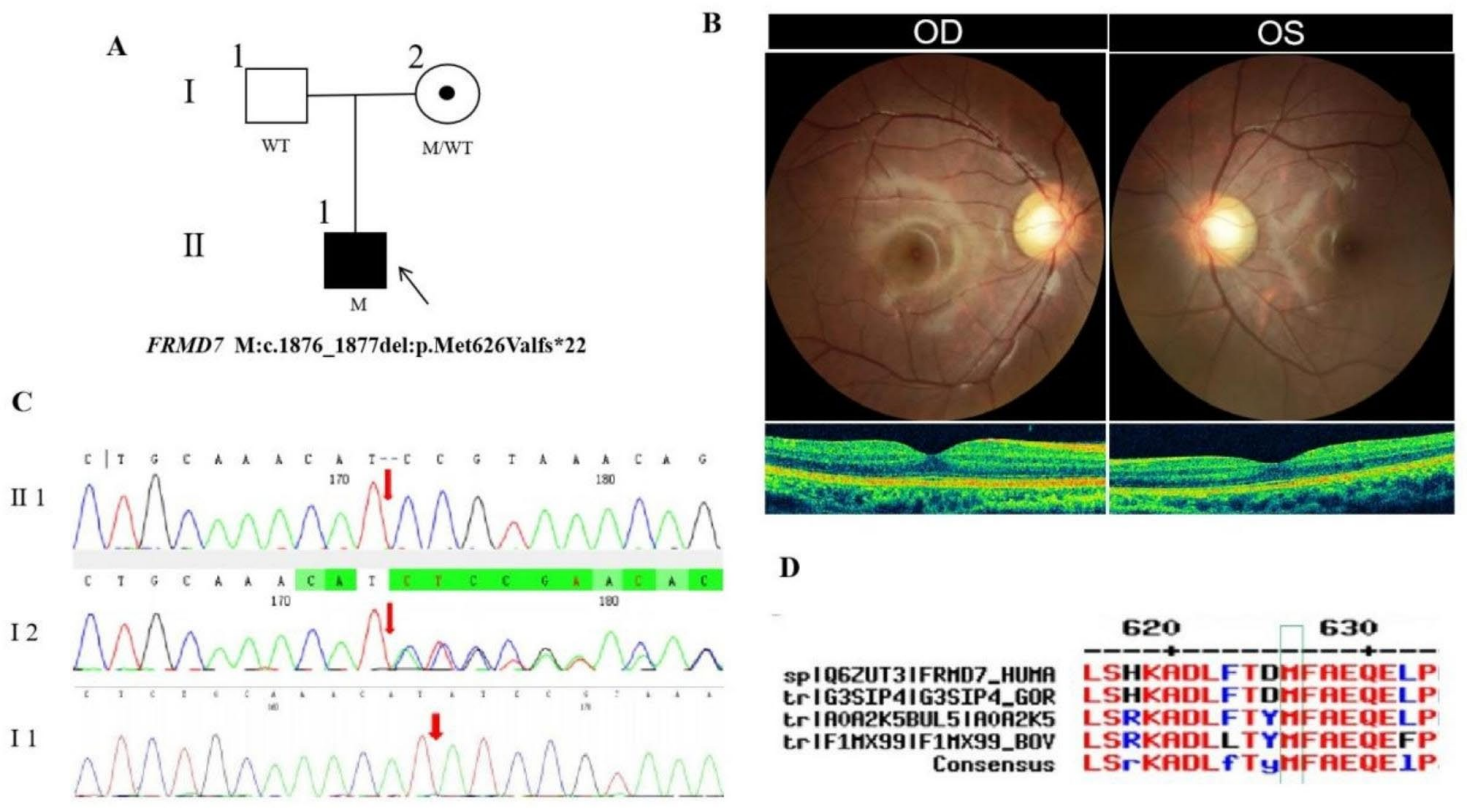
Sequence analysis and clinical examination of the family 4: (**A**) Pedigree of the family 4:The filled black symbol represents the affected member, a central dot denotes carriers, and the arrow denotes the proband. (**B**) The fundus of both eyes:the retina of both eyes were normal.The macular structure of both eyes was intact and the thickness was normal on OCT. (**C**) Sequence chromatograms of identified mutations. (**D**) The homology of amino acid sequences between human *FRMD7* and other species. The amino acid at position 626 is highly conserved among species, the mutated residue 626 is boxed and indicated

The proband, male, was 8 years old. His parents complained that the child had high myopia in both eyes for four years, and denied a family history of hereditary disease and consanguineous marriage history (Fig. 4A). He had horizontal nystagmus and BCVA of 0.6 + in both eyes. The anterior segment and fundus were normal (Fig. 4B). His mother had no nystagmus and BCVA of 0.8 + with high myopia in both eyes. The proband carried a hemizygous frameshift deletion variant c.1876_1877del(p.Met626Valfs*22) in the *FRMD7* gene on chromosome X and his mother was high myopia with the same heterozygous variant of the *FRMD7* gene by Sanger sequencing (Fig. 4C) (Table 2), suggesting co-segregation of genotype and clinical phenotype (PP1_Supporting). The frameshift deletion variant has not been previously reported and was also not detected in the East Asian Population Database (ExAC_ EAS) (PM2_Moderate). The frameshift deletion variant, c.1876_1877del, which was a 2-base deletion at site 1876–1877, resulted in a change from Methionine to Valine at residue 626 and generated the premature translational-termination codon at position 22 of the new reading frame, denoted p.Met626Valfs*22. What is more, the amino acid at position 626 was highly conserved among different species (Fig. 4D), indicating that the variant of this site is more likely to affect the structure and function of the *FRMD7* protein (PP3_Supporting). According to the ACMG guidelines, the frameshift variant c.1876_1877del (p.Met626Valfs*22) was pathogenic very strong (PVS1). The proband was finally diagnosed as X-linked recessive congenital nystagmus type 1 with early-onset high myopia. The hemizygous frameshift deletion variant c.1876_1877del (p.Met626Valfs*22) of the *FRMD7* gene was more likely the pathogenic variant of pedigree 4 according to ACMG guideline (PVS1 + PM2 + PP3 + PP1).

### Pedigree 5

**Fig. 5:**
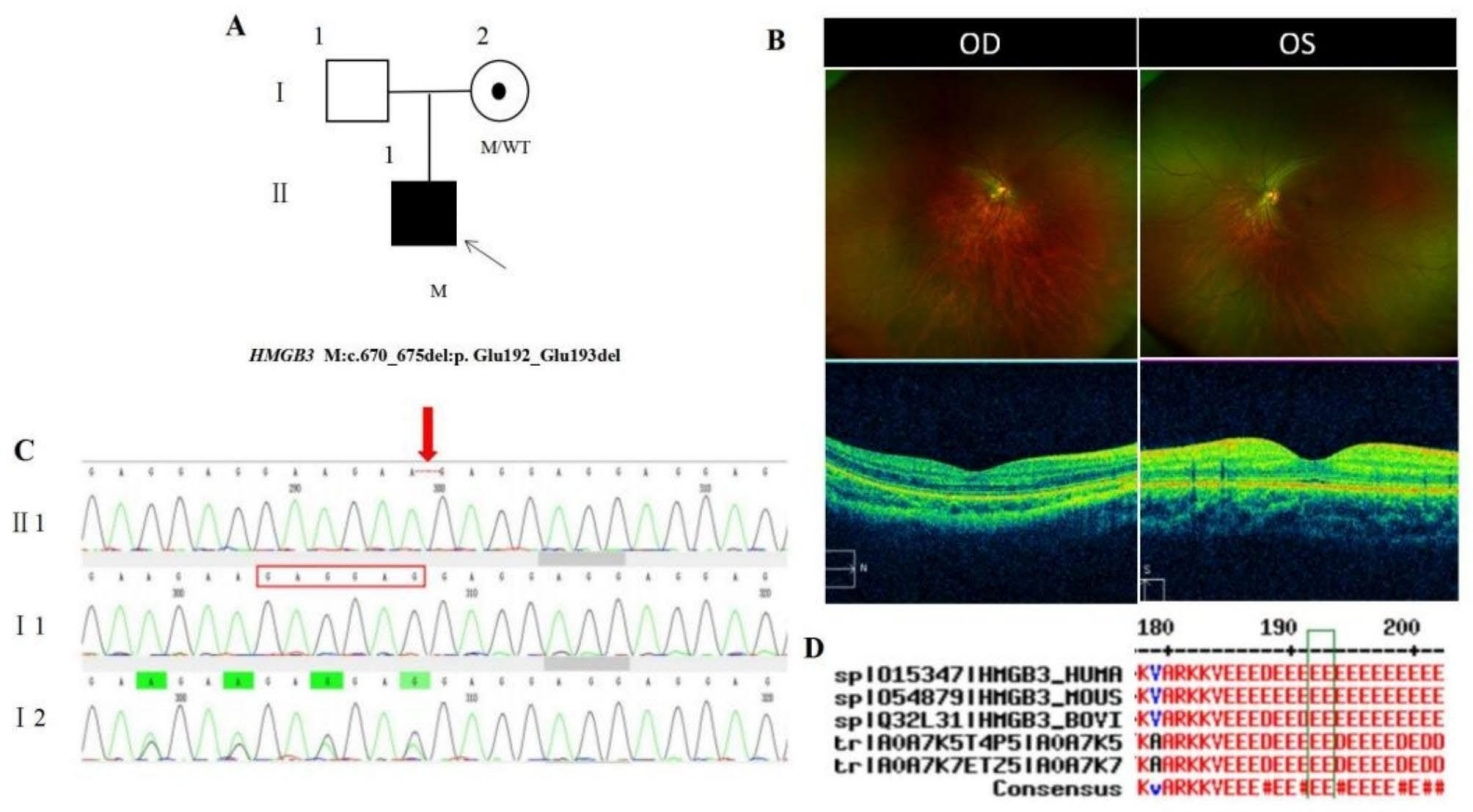
Sequence analysis and clinical examination of the family 5: (**A**) Pedigree of the family 5:The filled black symbol represents the affected member, a central dot denotes carriers, and the arrow denotes the proband. (**B**) The fundus of both eyes:Fundus tessellation with no reflection observed in macular fovea; the macular structure of both eyes was normal for OCT. (**C**) Sequence chromatograms of identified mutations (**D**) The homology of amino acid sequences between human *HMGB3* and other species. The amino acid at positions 192 and 193 are highly conserved among species, and the mutated residues 192 and 193 are boxed and indicated

The proband, a male, 12 years old, complained of poor vision in the right eye. His parents denied a family history and consanguineous marriage history (Fig. 5A). BCVA was 0.5 + on the right eye and 1.0 on the left eye (Table 1). He had mild ptosis and the anterior segment of both eyes was normal. Fundus tessellation with no reflection was observed in the macular fovea and OCT showed normal thickness and structure of the macular fovea (Fig. 5B). The proband carried a hemizygous inframe deletion variant c.670_675del(p.Glu192_Glu193del)was detected in the *HMGB3* gene on chromosome X and his mother was normal with the same heterozygous variant of the *HMGB3* gene by Sanger sequencing (Fig. 5C) (Table 2), suggesting co-segregation of genotype and clinical phenotype (PP1_Supporting).The deletion variant has not been previously reported and was also not detected in the East Asian Population Database (ExAC_ EAS) (PM2_Moderate), the amino acid at positions 192 and 193 were highly conserved among different species (Fig. 5D) (PP3_Supporting). The inframe deletion variant, c.670_675del,was a 6-base deletion at site 670–675, resulting in the deletion of encoded Glutamic acid at residues 192 and 193. According to the ACMG guidelines, the inframe deletion variant c.670_675del (p.Glu192_Glu193 del) was pathogenic very strong (PVS1). Finally, the proband was diagnosed as early onset high myopia in the right eye and ptosis in both eyes. The hemizygous inframe deletion variant c.670_675del (p.Glu192_Glu193 del) of the *HMGB3* gene was more likely the pathogenic variant of pedigree 5 according to ACMG guideline (PVS1 + PM2 + PP3 + PP1).

## Discussion

A study of which monogenic inherited retinal disorders cause myopia (and to what extent) may shed light on the processes underlying emmetropization, and those that drive refraction in a particular direction [26]. The dramatic rise in the incidence of myopia worldwide (around 30% in Europe [27] and over 70% in young people in some countries in East Asia [28]) has been identified as a public health problem, particularly in view of the increased risk of sight-threatening complications. Identifying the mechanisms that lead to myopia can inform the development of preventive interventions. Thus, further studies of the mechanisms and phenotypes of inherited retinal disorders, including the large proportion of X-related disorders, may provide insights not only into these disorders, but also into retinal physiology and pathophysiology more generally, including those associated with refractive errors, the most common ocular disorder.

In 2015, Li et al. demonstrated that *OPN1LW* gene causes syndromic high myopia and non-syndromic high myopia [20]. The variants in the *OPN1LW* gene can cause red and green visual dysfunction [29], which makes it easy to understand why the proband in pedigree 1 has both red and green visual dysfunction. Several studies have found that haplotype of the *OPN1LW* gene exhibits significant third exon skipping during pre-mRNA splicing, which is associated with the pathogenesis of high myopia [30, 31]. Qingjiong Zhang reported a case of high myopia with a frameshift mutation c.617_620dup (p.Phe208Argfs*51) in the fourth exon of the *OPN1LW* gene, inferring that such variant may trigger the mRNA decay and affect the clinical phenotype [20]. In addition to high myopia, ERG suggested a normal rod cell response and a moderately reduced cone cell response, similar to the clinical phenotype of the pedigree 1 proband in this study.

At the mRNA level, nonsense-mediated mRNA decay (NMD) prevents the expression of likely toxic truncated proteins by recognizing and decaying transcripts containing premature translational-termination codons (PTCs). NMD is efficient with PTCs located upstream of the last exon junction complex (EJC) [32]. This efficiency is governed by several rules such as 50nt rule and the last exon rule that are taken as canonical rules [33],which were not consistent with our data. PTCs of less than 50 nucleotides (nt) upstream of the last exon–exon junction will trigger NMD (the 50nt rule), and PTCs in the last exon of a gene or the terminal 50 bp of the penultimate exon do not trigger NMD (the last exon rule) and transcripts carrying such PTCs are transcribed at a level comparable to wild-type protein mRNA, resulting in deleterious effects of truncated proteins and leading to more severe clinical phenotype compared to those caused by 5′end PTCs [32, 33]. In this study, the *OPN1LW* gene has a total of 6 exons, and the predicted termination codon in this study occurs in exon 5, but not the last 50 base pairs of the penultimate exon, so NMD is likely to occur. Due to the nonsense-mediated mRNA decay mechanism, most mutated transcripts are eliminated before translation, and the disease symptoms arising therefrom are usually milder.

Approximately 15% of XLRP is caused by mutations in the *RP2* gene [34]. There are two mutation hotspots within the RP2 protein, the N terminus and TBCC domain where the novel *RP2* missense mutation c.513G > A(p.Trp171Ter) located [35]. Most *RP2* pathogenic mutations were located in the N-terminal parallel β helix domain, forming a tight hydrophobic core [36]. Disruption of the tightly packed hydrophobic core by mutations would normally destabilize the RP2 protein [37]. The mutation c.513G > A(p.Trp171Ter) was located in the first α helix and belongs to a long loop (aa 146–173), contacting the second and third β sheet of the hydrophobic core. This mutation may cause clashes with amino acids in 2/3 β sheet and destroy the hydrophobic core, leading to RP2 protein instability. The *RP2* gene contains 5 exons, and the presence of the predicted termination codon in exon 2 in this study triggers NMD, which allows most of the mutated transcripts to be eliminated before translation. Some previous studies support that NMD degradation depends on the position of the premature translation termination codons (PTCs),and the severity of the clinical manifestations correlates with the abundance of the PTCs, which increases in upstream truncating mutations resulting in fewer transcripts being retained [38].We used RT-qPCR to detect the expression of *RP2* gene transcript levels, and the results indicated that most of the mutated transcripts were eliminated before translation due to nonsense-mediated mRNA decay. Thus, it is not difficult to understand why the proband in pedigree 2 had a mild clinical phenotype. What is more, early macular involvement was found to be a unique clinical feature of *RP2* gene-related RP by Japanese scholars in 2010 [39]. It has been found as studied that approximately 50% of *RP2* gene-related RP patients are accompanied by high myopia. Therefore, in clinical practice, patients with X-linked inheritance, high myopia, early onset of central visual acuity impairment, and complicated with early-onset macular atrophy should first be considered for *RP2* gene variants and prioritized for screening of related gene variants.

Ocular albinism type I (OA1) is an X-linked genetic eye disease caused by mutations in the *GPR143* gene [40]. Male patients with OA1 usually present with severe vision loss, nystagmus, marked photophobia and varying degrees of strabismus [41]. Ocular examination reveals albinism-like fundus changes and macular dysplasia, but normal skin and hair color. The iris may be translucent in Caucasians but is usually absent in hyperpigmented Asian patients [42]. In the Chinese population, more than 80% of *GPR143* mutations are null mutations, while missense mutations account for only 18% [43]. The missense variant c.98G > T (p.Cys33Phe) of the *GPR143* gene in this study was located in the annular region within the lumen of melanosomes or lysosomes, and such variant locus was highly conserved across species, and by protein conformation, we speculated that such variant might lead to altered function by affecting the binding of the receptor. The proband in this study, in addition to the typical clinical phenotypes of OA1, was also complicated with high myopia. It has been found from some studies that, unlike the *TYR* gene-related albinism, *GPR143* gene-related albinism is more likely to be complicated with a short ocular axis [44]. In 2022, Pavan Verkicharla analyzed the refractive status of 618 albinos and found that high myopia being much more prevalent than hyperopia [45], suggesting that high myopia is also a common sign in albinism, and that if patients are complicated with high myopia, then, as in this case, albinism-like fundus changes are often less typical, so that OA1 is often misdiagnosed as idiopathic congenital nystagmus [46].

Idiopathic congenital nystagmus (ICN) is an ocular movement disorder and X-linked inheritance mode is more common, accounting for about 90% of cases [47], and the main pathogenic genes are *FRMD7* and *GPR143*, with variants in the *FRMD7* gene being more common [48]. Frameshift mutation always produces a premature stop codon and generates either a truncated protein or an abnormal mRNA that is degraded due to the NMD mechanism [49]. Previous studies have shown that the affected males carrying a premature terminated translation would have more severe clinical features than those males who carry with missense mutations [50],the *FRMD7* gene has 12 exons, and the termination codon predicted in this study occurs in the last exon, and this NMD transcriptional decay may not occur, resulting in a detrimental effect of the truncated protein. Theoretically, patients should present a more severe clinical phenotype, but it has also been found that there are transcripts containing PTCs that undergo NMD even though the PTC is in the last exon, while those aberrant transcripts that should undergo NMD (PTC at the upstream 3’ end) escape the NMD pathway and sometimes NMD does not completely decay all transcripts containing PTCs [32]. This makes it easy to understand that although the proband in this study had typical nystagmus and was complicated with early-onset high myopia, presenting a milder clinical phenotype.

The previous studies reported that ICN is usually complicated with varying degrees of hyperopia or mild myopia [51], with high myopia being relatively rare in ICN. In X-linked congenital idiopathic nystagmus pedigrees, the penetrance in female members can range from 30 to 100% [52, 53], and possible mechanisms for the occurrence of this incomplete penetrance include X chromosome inactivation, genetic modifiers, regulation of other genes and other non-genetic developmental influences (e.g. environment) on oculomotor development [54]. The key factor is X chromosome inactivation.The proband’s mother in this study had high myopia without nystagmus,which suggests that the variation in the *FRMD7* gene is responsible for high myopia.

*HMGB3* gene plays a key role in DNA replication, nucleosome assembly, and transcription [55]. In 2014, Alan F. Scott et al. detected the nonsense variant c.477_478insTA (p.Lys161Ilefs*54) in the *HMGB3* gene in an X-linked Colobomatous Microphthalmia Syndrome pedigree. This is the only study to date in which the *HMGB3* gene has been associated with ocular disease. The patient in this pedigree was an 8-year-old boy with a clinical phenotype of microcephaly, dysgnosia, and short stature. Ocular manifestations were microphthalmia, nystagmus, binocular ptosis, binocular subnasal coloboma of iridis, chorioretinal defect in the left eye, and esotropia in the right eye [56]. In this study, the proband carried the inframe deletion variant c.670_675del (p. Glu192_ Glu193del) of the *HMGB3* gene, which was not a nonfunctional variant. The proband only showed high myopia in the right eye and mild ptosis in both eyes, and there were no other systemic and ocular abnormalities. The association of different types of variants of the *HMGB3* gene with diverse clinical phenotypes remains to be further investigated in more clinical cases.

eoHM is often the early manifestations of retinal dystrophy in many patients, and clinicians tend to focus only on the change in myopia, while the true primary disease is easily overlooked, often resulting in misdiagnosis and underdiagnosis. In view of this, we remind clinicians that a detailed medical and family history should be performed in patients with eoHM to identify a certain genetic pattern through family history. This should be followed by a detailed ophthalmologic examination and the necessary systemic examination to look for clinical signs other than myopia, and if the patient is suspected of having a Mendelian disorder, whole-genome exome sequencing can be performed to further clarify the diagnosis.

In this study, we here identified five novel hemizygous variations in *OPN1LW, RP2, GPR143, FRMD7* and *HMGB3* genes that are associated with eoHM, extending the genotypic spectrums for eoHM.The variants in three of these genes (60%) produced PTC-containing mRNAs that triggered NMD, prevented the production of likely toxic truncated proteins, and played a key regulatory role in the phenotype of these diseases. Further analysis pathogenicity of these variations and the clinical phenotype of eoHM will assists ophthalmologists in assessment, diagnosing, and conducting genetic screening for eoHM but also provide a theoretical basis for eugenics for patient family members.

## Data Availability

The datasets generated and analyzed during the current study are available in the [Banklt] repository (BankIt (https://www.ncbi.nlm.nih.gov/nuccore/) ID: 2638435,2638433, 2,638,432, 2,638,430, 2,638,428).
